# Genotype-Dependent Jasmonic Acid Effect on *Pinus sylvestris* L. Growth and Induced Systemic Resistance Indicators

**DOI:** 10.3390/plants12020255

**Published:** 2023-01-05

**Authors:** Emilija Beniušytė, Ieva Čėsnienė, Vaida Sirgedaitė-Šėžienė, Dorotėja Vaitiekūnaitė

**Affiliations:** Laboratory of Forest Plant Biotechnology, Institute of Forestry, Lithuanian Research Centre for Agriculture and Forestry, Liepu St. 1, LT-53101 Girionys, Lithuania

**Keywords:** Scots pine, secondary metabolism, phenols, flavonoids, antioxidant enzymes, chlorophyll, carotenoids

## Abstract

Due to temperature changes, forests are expected to encounter more stress than before, both in terms of biotic factors, such as increased insect attacks, and abiotic factors, such as more frequent droughts. Priming trees to respond to these changes faster and more effectively would be beneficial. Induced systemic resistance (ISR) is a mechanism that is turned on when plants encounter unfavorable conditions. Certain elicitors, such as jasmonic acid (JA) are known to induce plants’ metabolic response. However, even though studies on ISR in herbaceous species are common and varied ISR elicitors can be used in agriculture, the same cannot be said about trees and forestry enterprises. We aimed to investigate whether JA used in different concentrations could induce metabolic changes (total phenol content, total flavonoid content, photosynthesis pigment content, antioxidant enzyme activity) in *Pinus sylvestris* seedlings and how this varies between different pine half-sib families (genotypes). After six weeks with a single application of JA, pine seedlings in several pine genetic families exhibited increased antioxidant enzyme activity, total phenol content and carotenoid content that correlated positively with JA concentrations used. Results from other genetic families were varied, but in many cases, there was a significant response to JA, with a noticeable increase as compared to the unaffected group. The impact on chlorophyll content and flavonoids was less noticeable overall. A positive effect on seedling growth parameters was not observed in any of the test cases. We conclude that JA can induce systemic resistance after a single application exogenously in *P. sylvestris* seedlings and recommend that the use of JA needs to be optimized by selecting appropriate concentrations.

## 1. Introduction

Induced systemic resistance (ISR) is a defense mechanism in plants by which a plant can protect itself from infection/infestation (pathogen, herbivore, etc.), usually without directly inhibiting or killing the invader, or it may help plants respond to stress [1,2,3,4,5]. This usually happens through the enhancement of plants’ natural defenses: enhancements in polyphenols, antioxidant enzyme activity, mechanical barriers, etc. [1,2,3]. Ait Barka et al. demonstrated that using bacteria on grapevine plants increased plant phenol production. Simultaneously, plants exhibited higher resistance to chilling [5]. ISR was also linked with reduced insect attack severity on pines [6] and reduced disease severity in *Pythium aphanidermatum* pathogen-infected cucumber plants [7]. 

Since systemic resistance induction is metabolically disadvantageous to the plant, it is only incurred when necessary. However, there is a time lag between attack or stressor impact and response induction, thus limiting the effectiveness of the plant’s response. Priming the plants by using certain ISR elicitors may help fill that gap. In short, the plant would be primed for future attacks or potential stress and would likely respond faster and more effectively [1,2,3,4,6,8,9].

There are several notable mechanisms that could be classified as induced systemic resistance. Firstly, a plant’s natural chemical defenses can be induced in the form of low molecular weight compounds such as phenolics, alkaloids, etc. [4]. One of the major classes of secondary metabolites is phenolic compounds. Phenols are frequently implicated in defense mechanisms against biotic stressors in woody plants, especially in conifer species. Larger amounts of phenolic compounds in plant tissues activate a specific defense response that is capable of stopping an invading pest or pathogen or may affect plants‘ response to stress [2,10]. Secondly, protein-based compounds, such as antioxidative enzymes may be produced [2,11]. It is emphasized that plants have an efficient antioxidant defensive system that helps them to counteract biotic and abiotic stress. Enzymatic antioxidants may include catalase (CAT), peroxidase (POX), ascorbate peroxidase (APX), glutathione reductase (GR) and others. [12,13,14]. Additionally, resistance could be in the form of structural barriers, natural enemy attraction or resource management [2,4]. Thaler et al. showed that using jasmonic acid as an ISR elicitor increased proteinase inhibitors and polyphenol oxidase levels. This correlated with decreased numbers of herbivore insects [11]. Similarly, Zas et al. report that after methyl jasmonate (MeJA) application, three conifer species showed enhanced non-volatile resin and polyphenolics levels. The former constitutes a structural–mechanical defense, and the latter a chemical defense [4]. It is worth noting that it is quite usual that all the ISR mechanisms work in parallel [2]. 

The antioxidant defensive system is composed of both enzymatic and non-enzymatic mechanisms. Non-enzymatic antioxidants include ascorbate, glutathione and photosynthesis pigments, such as carotenoids [12,13,14]. Additionally, plant response indirectly could be indicated through other photosynthesis pigments (i.e., chlorophylls) [15,16,17]. 

ISR has been studied in herbaceous species and recently in woody plants as an eco-friendly means of enhancing plant resistance. While ISR elicitors were shown to work and have been applied commercially in agriculture for some time, their positive affect on herbaceous species may not directly translate to trees, as these models have several major differences (i.e., lifespans, different organs, etc.) [2,4]. 

ISR research is especially important in the face of recent abiotic and biotic changes in the environment. Between 1995 and 2010, plant infectious diseases (fungal pathogens mostly) have increased 13-fold, and this trend is expected to grow [18]. This is most likely due to temperature shifts (i.e., warmer winters, longer and warmer summers). Thus, ISR elicitors could be used to limit pesticide applications in commercial forestry and associated enterprises. Recent temperature changes also affect insect populations and may enhance their active range, lengthen feeding periods, etc. Global changes will also likely impact drought and other unfavorable event frequency [2,19,20,21,22]. All of this may affect trees [20]; thus, ISR could be used to alleviate some of that stress and limit the damage to forest ecosystems and forestry services in a variety of ways. 

ISR works through jasmonate and ethylene metabolic pathways [1,2,3]. It has been shown that in trees, several molecules can serve as ISR elicitors, namely jasmonic acid (JA), salicylic acid, MeJA, chitosan, oxalic acid and ethylene. Alongside these, various bacteria and fungi can also elicit ISR-related responses [2]. 

Jasmonic acid itself and related jasmonates are plant growth regulators linked with stress mediation and, more specifically, resistance to pathogen attacks and herbivores [23,24,25]. As reported by Zas et al. [4], Thaler et al. [11], Sabbagh et al. [7] and others, jasmonates are an effective ISR elicitor that can help with plant response to abiotic and biotic stressors. Jasmonates were even shown to work in several studies on pines and other conifers [4,8,24,25,26,27]. However, while previous studies have examined ISR‘s importance for plant resistance mechanisms to biotic and abiotic stress, there is a gap in analyzing its impact on conifer species. To improve our understanding of tree resistance mechanisms, we aimed to evaluate how Scots pine genetic properties may affect this. 

Based on these data, we hypothesized that jasmonic acid could induce systemic resistance in *Pinus sylvestris* seedlings after a single application and affect their growth and that this would likely be genotype dependent. 

Results showed that JA did induce systemic resistance in Scots pine seedlings, but it was dependent on pine genotype and JA concentration used. JA did not positively affect the pine trees’ initial growth.

## 2. Results

### 2.1. Growth Parameters

**Figure 1:**
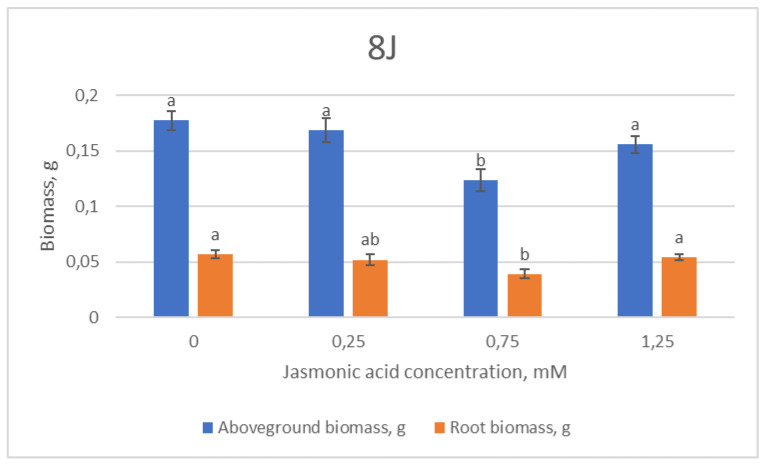
Means (g) ± SE of above-ground and root biomass of Scots pine half-sib family 8J, measured 6 weeks after sowing. Data significance was calculated using the Kruskal–Wallis H test for ranks and post hoc Dunn’s test for pairs (*p* < 0.05). Different letters next to different colors indicate significant differences between the groups, sprayed with different concentrations of jasmonic acid.

When evaluating morphometric growth parameters of the 8J family in different experimental variants depending on the jasmonic acid (JA) concentration, it was found that JA had an overall negative effect or no effect (Figure 1).

The 8J group treated with 0.75 mM JA concentration presented the significantly lowest overall morphometric and biomass values (Figure 1). Subsequently, when a higher concentration of JA was used, the values leveled out to control-group levels in all measured morphometric parameters. 

**Figure 2:**
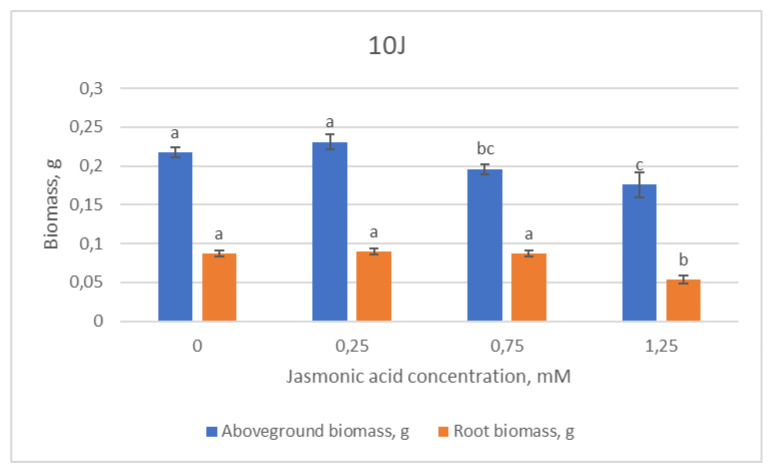
Means (g) ± SE of above-ground and root biomass of Scots pine half-sib family 10J, measured 6 weeks after sowing. Data significance was calculated using the Kruskal–Wallis H test for ranks and post hoc Dunn’s test for pairs (*p* < 0.05). Different letters next to different colors indicate significant differences between the groups, sprayed with different concentrations of jasmonic acid.

The morphometric values of the 10J half-sib family showed a tendency to decrease with an increase in JA concentration used, both in shoot length, longest needle length and root length, as well as above- and below-ground biomass (Figure 2).

**Figure 3:**
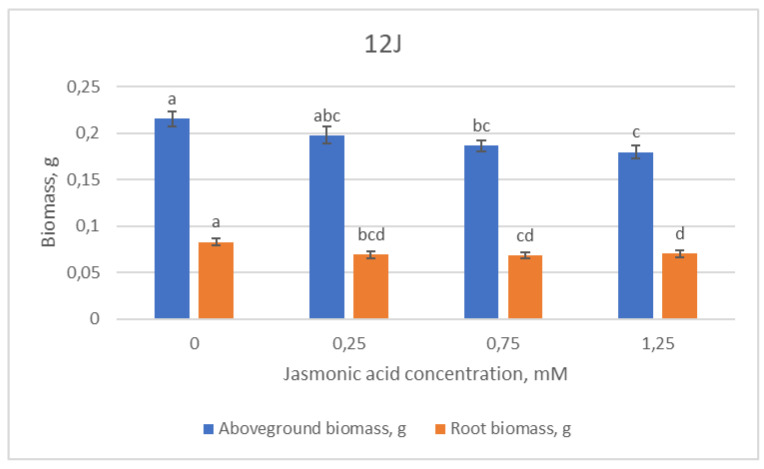
Means (g) ± SE of above-ground and root biomass of Scots pine half-sib family 12J, measured 6 weeks after sowing. Data significance was calculated using the Kruskal–Wallis H test for ranks and post hoc Dunn’s test for pairs (*p* < 0.05). Different letters next to different colors indicate significant differences between the groups, sprayed with different concentrations of jasmonic acid.

The morphometric data of the 12J half-sib family showed mixed tendencies for every measured parameter (Figure 3). The values of shoot length presented no significant differences between groups, whilst the longest needles were found in the control group and decreased steadily with an increase in JA concentration. The length of the main root decreased with increasing JA concentration as well; however, in the highest (1.25 mM) concentration group, it leveled out to control group levels.

Biomass values of the 12J family also showed a tendency to decrease with increasing JA concentration (Figure 3). The highest values of above-ground and root biomass were recorded in the control group, whilst the lowest were in the group treated with 1.25 mM JA concentration.

The morphometric data of the 16J p”ne h’lf-sib family did not show any statistically reliable difference between the groups, except for the decrease in the length of the main root in the group treated with the lowest (0.25 mM) JA concentration.

**Figure 4:**
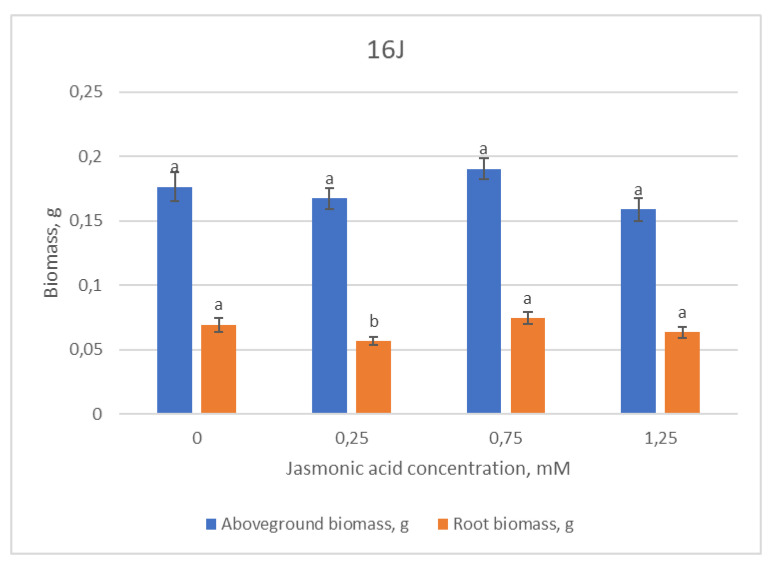
Means (g) ± SE of above-ground and root biomass of Scots pine half-sib family 16J, measured 6 weeks after sowing. Data significance was calculated using the Kruskal–Wallis H test for ranks and post hoc Dunn’s test for pairs (*p* < 0.05). Different letters next to different colors indicate significant differences between the groups, sprayed with different concentrations of jasmonic acid.

No significant differences between groups were recorded while evaluating the 16J family’s above-ground biomass data (Figure 4). Meanwhile, root biomass significantly decreased only after treatment with 0.25 mM JA concentration, as noted in the root length as well. 

When examining the length of the shoot in family 31J, it is possible to single out the group treated with the concentration of 0.75 mM of JA, where the length of the shoot was found to be statistically lower, compared to other groups. The longest main roots were recorded in the control group and the group treated with the highest JA concentration. Meanwhile, the morphometric data of the longest needle did not show a clear trend.

**Figure 5:**
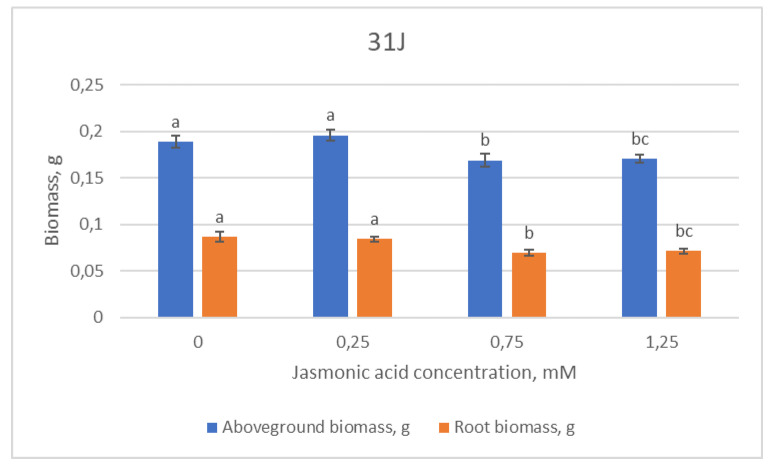
Means (g) ± SE of above-ground and root biomass of Scots pine half-sib family 31J, measured 6 weeks after sowing. Data significance was calculated using the Kruskal–Wallis H test for ranks and post hoc Dunn’s test for pairs (*p* < 0.05). Different letters next to different colors indicate significant differences between the groups, sprayed with different concentrations of jasmonic acid.

The highest values of aboveground and root biomass among the 31J family groups were found in the control and 0.25 mM JA groups and statistically decreased with the increasing JA concentration further on (Figure 5).

### 2.2. Secondary Metabolites

**Figure 6:**
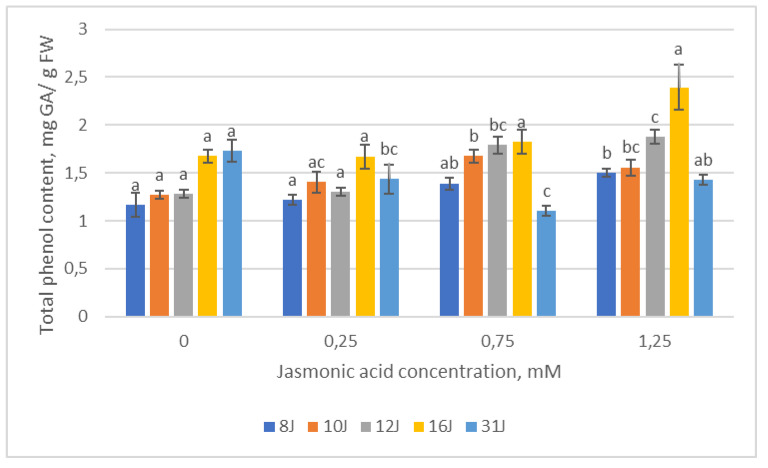
Means (mg/g) ± SE of total phenolic compounds of five half-sib families of Scots pine, assessed 6 weeks after sowing. Data significance was calculated using the Kruskal–Wallis H test for ranks and post hoc Dunn’s test for pairs (*p* < 0.05). Different letters next to different colors indicate significant differences between the groups, sprayed with different concentrations of jasmonic acid.

During the study, the total amount of phenolic compounds (TPC) accumulated in the needles of every experimental group was evaluated (Figure 6). TPC synthesized in the needles of the 8J and 12J half-sib families increased with the increasing JA concentration. In the 10J family, the total amount of phenols also increased with increasing JA concentration; however, the amount decreased at the highest (1.25 mM) JA concentration. In the 16J family, the highest TPC concentrations among all families were displayed; however, there were no significant differences between families’ experimental groups. Meanwhile, TPC synthesized in the needles of the 31J family seedlings decreased with the increase in JA concentration up to the highest (1.25 mM) concentration, where a statistical increase was recorded. 

8J, 12J and 16J family data displayed no significant differences in TFC concentrations between experimental groups. In the 10J family, TFC concentration seemed to decrease with increasing JA concentration, whereas the amount of flavonoids accumulated in the 31J family seedlings was the lowest out of all families and did not display significant tendencies depending on JA concentration.

A strong positive significant correlation (here and later R > 0.6, *p* < 0.05) between TPC and TFC was determined in 10J and 12J half-sib families after treatment with the 0.25 mM and 1.25 mM JA concentrations. Correlation analysis revealed that with the increase of secondary metabolites, chlorophyll a and chlorophyll b also increased in half-sib families 31J, 8J and 10J after treatment, with the lowest and the highest JA concentration. Chlorophyll a content in the 10J, 12J and 16J half-sib families remained statistically identical, regardless of JA concentration used. The concentration of chlorophyll a in 8J family experimental groups was higher in the control group than in the treated groups, regardless of concentration. Meanwhile, the lowest amount of chlorophyll a among all half-sib families was recorded in the 31J family group that was treated with 0.25 mM JA concentration.

The concentration of chlorophyll b synthesized in the 10J, 12J and 16J groups showed no significant differences, regardless of JA concentration used. In the 8J family, the concentration of chlorophyll b exhibited significant differences only between the control group and the JA treated groups, and it positively correlated with chlorophyll a in all treatments with JA. The same correlation results between chlorophyll a and chlorophyll b were obtained after treatment with all JA solutions. Meanwhile, in the 31J family, the group treated with the 0.25 mM JA concentration stood out the most and had the statistically lowest chlorophyll b concentration of all measured groups. Moreover, both chlorophyll a and b amounts showed the same tendencies. 

No statistically significant differences in the concentration of carotenoids were observed among the 12J and 31J experimental groups, regardless of the JA concentration used. Correlation analysis showed that the concentration of carotenoids statistically significantly decreased with the increase of chlorophyll a and chlorophyll b in 10J and 16J half-sib families after treatment with the lowest (0.25 mM) and the highest (1.25 mM) JA concentrations, respectively. In the 8J family, the concentration of carotenoids seemed to significantly increase in the group treated with the highest JA concentration. Meanwhile, in the 10J family, the carotenoid concentration decreased with increasing JA concentration, except for the highest (1.25 mM) JA concentration, where carotenoid concentration is observed to be statistically comparable with that of the control group. In the 16J family, carotenoid concentration showed no linear dependency on JA concentration.

### 2.3. Antioxidative Enzymes

**Figure 7:**
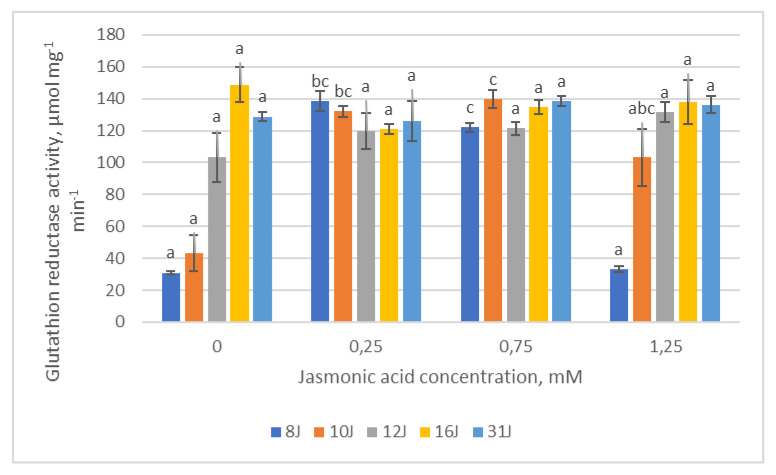
Means (µmol mg^−1^ min^−1^) ± SE of glutathione reductase (GR) activity in five half-sib families of Scots pine, assessed 6 weeks after sowing. Data significance was calculated using the Kruskal–Wallis H test for ranks and post hoc Dunn’s test for pairs (*p* < 0.05). Different letters next to different colors indicate significant differences between the groups, sprayed with different concentrations of jasmonic acid.

During this study, the dependence of the activity of antioxidant enzymes (CAT, POX, APX, GR) on the concentration of JA was evaluated. In 12J, 16J and 31J families, the activity of GR had no significant differences among experimental groups, regardless of JA concentration (Figure 7). Moreover, a statistically significant positive correlation was obtained between GR and TPC, TFC and chlorophylls in the 31J half-sib family after treatment with the lowest JA concentration. In the 8J and 10J family groups, GR activity peaked in groups treated with 0.25 mM and 0.75 mM JA concentrations and dropped again at the highest JA concentration. Correlation analysis revealed that the increase of GR could be related to the increase of TFC and TPC in groups treated with 0.25 mM and 0.75 mM JA concentrations, respectively.

**Figure 8:**
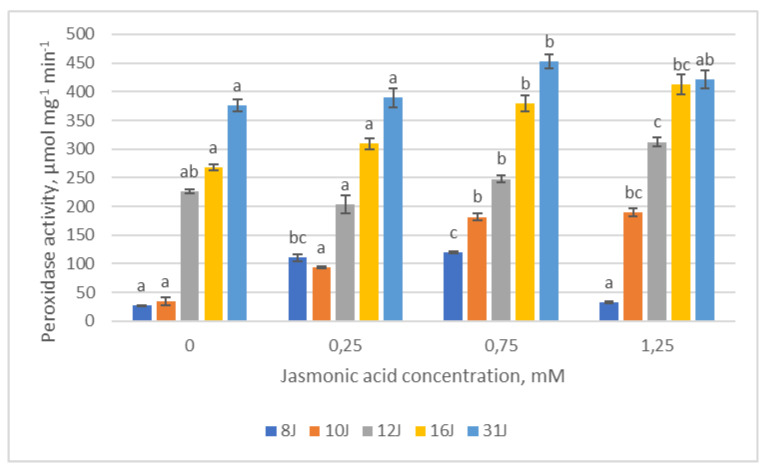
Means (µmol mg^−1^ min^−1^) ± SE of peroxidase (POX) activity in five half-sib families of Scots pine, assessed 6 weeks after sowing. Data significance was calculated using the Kruskal–Wallis H test for ranks and post hoc Dunn’s test for pairs (*p* < 0.05). Different letters next to different colors indicate significant differences between the groups, sprayed with different concentrations of jasmonic acid.

The changes in GR activity positively correlated with POX activity in 8J and 31J half-sib families after treatments with the lowest (0.25 mM) and highest (1.25 mM) JA concentrations. POX in the needles of 10J, 12J and 16J families showed a tendency to increase with increasing JA concentration (Figure 8). In the 8J and 31J families, a similar tendency was established; however, POX activity would decrease once the highest concentration of jasmonic acid was applied, as was similarly noted with GR in the 8J family. 

Correlation analysis revealed that POX positively correlated with TPC, chlorophyll a and chlorophyll b in the 12J and 16J families and with TFC in the 31J half-sib family after the treatments with the lowest (0.25 mM) JA concentration. Significant negative correlations were determined between POX and TPC, chlorophyll a and chlorophyll b in the 16J half-sib family in the group treated with the highest (1.25 mM) JA concentration.

**Figure 9:**
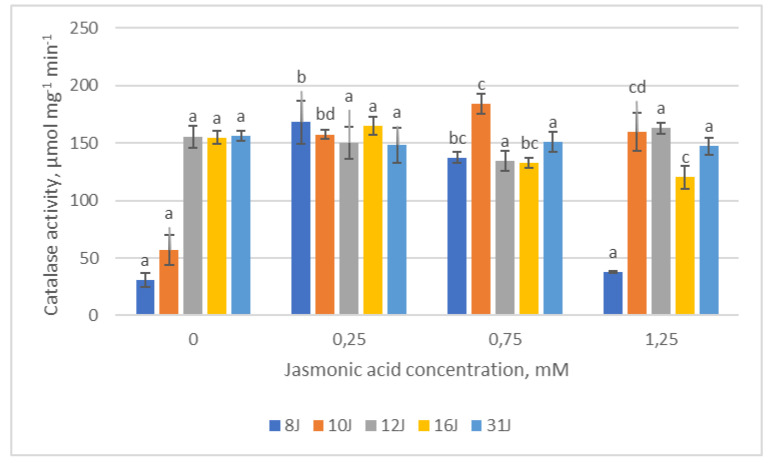
Means (µmol mg^−1^ min^−1^) ± SE of catalase (CAT) activity in five half-sib families of Scots pine, assessed 6 weeks after sowing. Data significance was calculated using the Kruskal–Wallis H test for ranks and post hoc Dunn’s test for pairs (*p* < 0.05). Different letters next to different colors indicate significant differences between the groups, sprayed with different concentrations of jasmonic acid.

Catalase (CAT) activity among the 12J and 31J family groups remained statistically identical, regardless of JA concentration (Figure 9). The highest CAT activity within the 8J experimental groups was recorded in the group treated with the lowest (0.25 mM) JA concentration. Correlation analysis revealed that CAT positively correlated with POX, APX and GR in the 8J experimental group treated with the lowest (0.25 mM) JA concentration. Moreover, the analysis showed that TPC, chlorophylls and CAT activity decreased in the 12J family after treatment with the highest (1.25 mM) JA concentration. In the 10J family, the CAT activity showed a significant tendency to increase with increasing JA concentration and dropped down in the group affected with the highest concentration (1.25 mM). In the 16J family, the opposite trend was observed, CAT activity decreased with increasing JA concentration. However, a positive significant correlation was observed between CAT and TFC in the 16J family after treatment with the lowest JA concentration.

**Figure 10:**
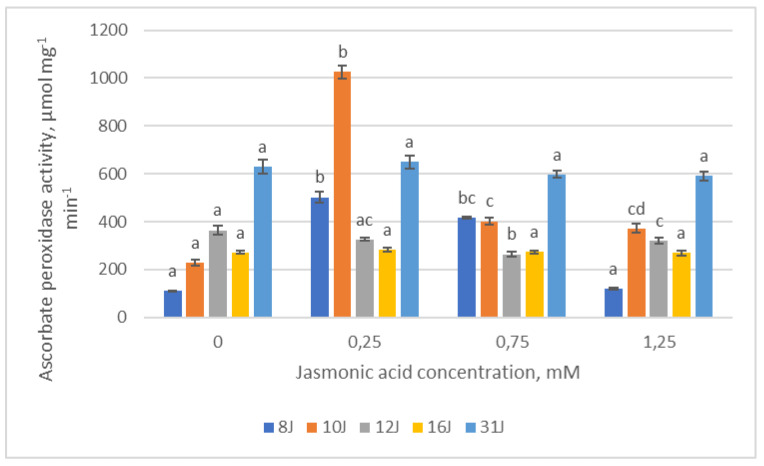
Means (µmol mg^−1^ min^−1^) ± SE of ascorbate peroxidase (APX) activity in five half-sib families of Scots pine, assessed 6 weeks after sowing. Data significance was calculated using the Kruskal–Wallis H test for ranks and post hoc Dunn’s test for pairs (*p* < 0.05). Different letters next to different colors indicate significant differences between the groups, sprayed with different concentrations of jasmonic acid.

In the experimental groups of the 16J and 31J families, ascorbate peroxidase (APX) activity remained statistically identical, regardless of JA concentration (Figure 10). The highest APX activity among the 8J and 10J family groups was recorded in the groups treated with the lowest (0.25 mM) JA concentration. Meanwhile, in the 12J family, APX activity had a tendency to decrease with increasing JA concentration, except in the 1.25 mM group, where it levels out. Correlation analysis showed strong positive dependence between APX, POX and GR in all five Scots pine half-sib families after treatment with the highest JA concentration. The changes in APX activity positively correlated with TPC in the 16J half-sib family treated with the lowest (0.25 mM) and highest (1.25 mM) JA concentration. The same dependence was determined between APX and TPC in the 31J half-sib family after the treatments with the 0.75 mM JA concentration. Moreover, a strong negative correlation in the 12J and 16J families treated with 0.25 mM and 0.75 mM JA concentration was observed between APX activity, CAR and chlorophylls. 

## 3. Discussion

Compounds from the jasmonate group, such as jasmonic acid (JA) and methyl jasmonate (MeJA), are considered as plant growth regulators, which take part in the plant’s response to environmental stressors. When used exogenously, they can cause a physiological, biochemical and genetic response in plants [26,28,29,30,31,32]. 

Studies show that jasmonates could potentially be used to induce natural protective mechanisms within the plant, which would potentially allow less pesticides to be used in both agriculture and forestry [26,32], as well as help plants face other stressful situations (e.g., drought) [33,34]. MeJA, which is a derivative ester of jasmonic acid, is the most widely studied jasmonate [26]. 

Results from previous studies show that Norway spruce can exhibit induced systemic resistance (ISR) due to the exogenous application of MeJA. ISR was observed after 8–30 days after application ([35] and references therein). Krokene et al. showed that Scots pine exhibited enhanced resistance to pathogenic fungi *Ceratocystis polonica* after MeJA application. Specifically, the size (length) of necrotic lesions decreased by 51% [35]. Similarly, Kozkowski et al. noted that Norway spruce exhibited enhanced (by 75%) resistance against *Pythium ultimum* pathogenic fungi. It was observed that by applying 3 mM JA on spruce seeds, *Hylobius abietis* attacks were reduced by 62.5% [36]. 

JA can also affect the growth of Norway spruce. Heijari et al. showed that exogenous MeJA application not only reduced *Hylobius abietis* attacks but also negatively impacted Norway spruce growth, with reduced root and shoot biomass [37]. A similar effect was also noted in our work in some of the tested Scots pine genetic families. Martin et al. demonstrated that JA can affect *Pinus pinaster* tree epicotyl height negatively [38]. Another study showed that low JA concentrations increased root hair numbers [39]. In our experiment, neither of the three tested JA concentrations affected pine seedling growth positively. Furthermore, in some of the tested pine genetic families, vegetative growth parameters were statistically lower as compared to the control group. Thus, all in all, the JA application had either no effect or a statistically negative effect on Scots pine vegetative growth parameters during the studied period. It is possible that this negative effect could be resolved in the long run, as was noted in two-year-long ex vitro trials with both Norway spruce and Scots pine [4].

During our work, JA impact on total phenol, total flavonoid, chlorophyll a and b and carotenoid content was measured. Flavonoids are a class of phenols that were shown to function as antioxidants [26,40]. During our experiment, in some cases TPC and TFC correlated positively with enhanced antioxidative enzyme activity. Usually, an increase in phenol content is often linked with ISR and enhanced plant resistance, as is an increase in total flavonoid content [15]. During this experiment, we observed that in four of the five tested pine genetic families, the total phenol content had increased proportionally to JA concentration. An analog impact on TFC was not observed, even though in some families and with certain JA concentrations, TPC positively correlated with TFC; thus, this potentially indicates that the effect on TPC was not always related to flavonoid content. Zas et al. demonstrated that a single MeJA application did not have a statistically significant impact on phenol content in *Pinus sylvestris* trees. However, during the same experiment, an effect was noted after a second MeJA application, with a decrease in polyphenol content [4]. In a previously mentioned study, in which MeJA created a resistance response in spruce trees, an increase in polyphenol parenchymal cell number was also observed [35].

It is known that both carotenoids and chlorophylls have a secondary antioxidant function [41,42]. It is also noteworthy that photosynthesis pigment content is linked with general vitality and health of the plant [15]. During our study, we observed no tendencies in photosynthesis pigment content in common between genetic families. In some tested families, JA application had no effect, whereas in others it was negative. In some groups, a negative correlation between chlorophylls and carotenoids was observed. Scots pine half-sib family 8J exhibited an increase in carotenoid content and chlorophyll *a* and *b* content. In most cases photosynthesis pigment content increased proportionally to JA concentration used. Gould et al. demonstrated that after 1 mM MeJA application, *Pinus radiata* trees exhibited enhanced chlorophyll synthesis as compared to the control group; however, when the concentration was increased over 1 mM, the effects turned negative [43]. Heijari et al. also showed that low MeJA concentration can positively affect photosynthesis activity [37].

Antioxidant enzymes, such as peroxidase (POX), glutathione reductase (GR), catalase (CAT) and ascorbate peroxidase (APX), can also be used as ISR indicators, and in our work some of the tested enzyme activity correlated positively amongst each other and, as previously mentioned, with TPC. Higher amounts of these enzymes are linked with enhanced antioxidative resistance and hence with plants’ resistance to stress [28,44,45,46,47]. During our study, different JA concentrations were shown to affect antioxidant enzyme amounts in several of the tested pine families. It was shown that four tested antioxidant enzymes significantly increased proportionally to JA concentrations in families 8J and 10J. Guan and Scandalios noticed that after JA application on maize, CAT activity increased [48]. In another study, POX and polyphenol oxidase enzyme activity increased after MeJA application in *Pinus radiata* stems [43]. Similarly, JA application on *Brassica rapa* plants resulted in increases in photosynthesis pigment content (both chlorophyll and carotenoids) as well as enhanced antioxidative activity [33].

Overall, a similar negative jasmonate effect on growth parameters and a positive effect on secondary metabolism was noted in a review by Moreira et al. [26]. It is worth noting that the five tested *Pinus sylvestris* half-sib families exhibited different responses to JA application. Previous related studies show that different genotypes from the same species can exhibit varied production of secondary metabolites, antioxidant activity and growth [15,16]. Similar variations were observed in Norway spruce as well [42]. Bandurska et al. and Ahmad Lone et al. also discovered that plant genotype affects their response to JA application [33,49].

## 4. Materials and Methods

### 4.1. Seeds

*Pinus sylvestris* seeds were collected in a seed plantation in 2021 and then kept at +4 °C in moisture-proof bags. Seeds from five half-sib families were used: 8J, 10J, 12J, 16J and 31J. Families were chosen at random. At the beginning of the experiment, seeds were husked and kept at room temperature for 24 h before sowing. 

### 4.2. Sowing

Pine seeds were sown in germination cassettes, 25 mL of SuliFlor SF2 peat substrate per pot. (pH of 5.5–6.5) (Sulinkiai, Lithuania). One pine seed was sown in the center of each pot and a few millimeters of soil was sprinkled on top. The seedlings were grown in a greenhouse under natural light and temperature conditions for 6 weeks during the summer of 2022 (June–July). 

### 4.3. Treatment with Jasmonic Acid

Three weeks after the start of the experiment, the seedlings of each genetic family were divided into four experimental groups and sprayed with 0.25 mM, 0.75 mM and 1.25 mM jasmonic acid solutions in 0.1% acetone (in water). Control groups were treated with 0.1% acetone. Seedlings were sprayed with a manual sprayer until the shoot surface was completely covered [19,20]. For each group, 600 mL of the required solution was used. Treated groups were kept separately for three days to avoid cross-contamination and then transferred to the same growth chamber to maintain uniform growth conditions. 

### 4.4. Collection of Samples

Six weeks after sowing, Scots pine seedling measurements of above-ground and root lengths, as well as longest needle length, and above-ground and root biomass were carried out (at least 20 individuals per group). For further analysis, a sample of 1.15 g of shoot mass was taken from each variant replicate from at least 8 individual shoots, for a total of 60 samples (5 genetic families × 4 groups × 3 replicates). Each biological replicate was also measured thrice (i.e., three technical replicates). 

### 4.5. Preparation of Extracts for Secondary Metabolite and Photosynthetic Pigment Analysis

Quantification of photosynthetic pigment (chlorophyll a and b, carotenoids), total phenol (TPC) and total flavonoid content (TFC) was performed spectrophotometrically using SpectroStar Nano microplate reader (BMG Labtech, Offenburg, Germany) and 96-well microplates. A total of 100 g of pine needle biomass was homogenized in a pestle and mortar and poured over with 2 mL of 80% (*v/v* in water) ethanol. The samples then were centrifuged for 30 min, 21,910× *g*, +4 °C using a Hettich Universal 32R centrifuge (Andreas Hettich GmbH & Co. KG, Tuttlingen, Germany). The supernatant was then removed and used further. 

### 4.6. Quantification of Chlorophyll a, b and Total Carotenoids

Analyses were performed with fresh extract in the dark to minimize component degradation by light. The absorption of the extract was measured at the wavelengths of 470 nm, 648 nm and 664 nm. The concentration of chlorophyll *a*, *b* and total carotenoids was calculated using the formulas produced by Lichtenthaler and Buschmann [50]:

C(chl *a*) = (13.36 × A_664_) − (5.19 × A_648_) (1)

C(chl *b*) = (27.43 × A_648_) − (8.12 × A_664_) (2)

C(*carotenoids)* = (1000 × A_471_ − 2.13 × C(chl *a*) − 97.64 × C(chl *b*))/209 (3)

where A is the absorption of the extract at the respective wavelength; C(chl *a*), C(chl *b*) and C(*carotenoids*)—concentrations of alpha and beta chlorophyll and total carotenoids in the extract (μg/mL).

Photosynthetic pigment concentration in a gram of fresh needle biomass is calculated according to the following formula:

(4) $${\bar{C}}_{X}\;(\text{µ}g/g)=(C\;\times \;V\;\times \;W)/M$$

where ${\bar{C}}_{X}\;$is the concentration of pigments in fresh needle biomass (μg/g); C—concentration of pigments in the extract (μg/mL); V—volume of crude extract (ml); W—dilution of crude extract (units); M—weight of extracted biomass (g).

### 4.7. Determination of TPC

TPC was determined using the Folin–Ciocalteu reagent according to a modified methodology [51]. Samples were prepared in 96-well microplates, where 10 μL of the extract was mixed with 50 μL of Folin–Ciocalteu reagent (1:9 *w*/*v* in water) (VWR International GmbH, Vienna, Austria). Microplate contents were mixed well and incubated for 5 min, then 40 μL of 10% Na_2_CO_3_ was added. After 1 h of incubation in the dark, sample absorption at a wavelength of 725 nm was measured.

Gallic acid (>98%, Carl Roth GmbH + Co. KG, Karlsruhe, Germany) was used for the calibration curve: y = 0.0241x + 0.0206 (R^2^ = 0.9912).

TPC is expressed as micrograms of gallic acid equivalent in one gram of fresh mass (mg/g):

Concentration (mg/g) = (C × V)/m(5)

where C is the concentration obtained from the calibration curve (mg/mL); V—extract volume (ml); m—weight of fresh biomass extracted (g).

### 4.8. Determination of TFC

TFC was estimated based on the formation of a flavonoid-Al(III) complex [52]. Sample absorption at a wavelength of 415 nm was measured. Samples were prepared in 96-well microplates, where 20 μL of extract was mixed with 200 μL of reaction buffer (60 μL of absolute ethyl alcohol, 10 μL of 10% (w/v in water) aluminum chloride (99% purity) solution, 10 μL of 1 M potassium acetate (99% purity) and 120 μL of distilled water (dH_2_O)).

Quarcetin (>98%, Cayman Chemical Company, Ann Arbor, USA) was used for the calibration curve: y = 0.0366x + 0.0122 (R^2^ = 0.995).

TFC is expressed as micrograms of the quercetin equivalent in one gram of fresh biomass (mg/g):

Concentration (mg/g) = (C × V)/m(6)

where C is the concentration obtained from the calibration curve (mg/mL); V—extract volume (ml); m—weight of raw biomass extracted (g).

### 4.9. Sample Preparation for Antioxidant Enzyme Tests

Fresh biomass (~0.3 g) was triturated with liquid nitrogen and poured over with 5 mL of extraction buffer solution (preparation is given in Table 1). The samples were then centrifuged (1 h, 16,090× *g*, +4 °C). After centrifugation, the supernatant was prepared for total protein (PROT) and catalase (CAT) analyses.

For the analysis of ascorbate peroxidase (APX), guaiacol peroxidase (POX) and glutathione reductase (GR), separation of the extract supernatant was carried out through Sephadex G-25 (Column PD-10, Cytiva, Gillingham, UK) columns on ice [31]. All reactions for the protein and enzyme extraction were performed on ice [53]. 

#### 4.9.1. Total Protein (PROT)

The chosen method for determining the total protein concentration is based on the peptide bond reaction between the reagent and the investigated proteins, when Cu^2+^ ions are reduced to Cu^+^ [55]. For protein analyses, 20 μL of crude extract is added to 180 μL of Biuret reagent (Table 1) and 20 μL of Folin–Ciocalteau reagent (1:9 *w/v*). Samples were then incubated at room temperature for 50 min. Then, the absorption is measured at 660 nm. 

After measuring the absorption of the standard—BSA (Bovine Serum Albumin) (>98%, Sigma-Aldrich), a calibration curve was constructed, from which the following equation was obtained: y = 0.1003x − 0.0462 (R^2^ = 0.9747). 

The total amount of protein is expressed as micrograms of the BSA equivalent in one milliliter of crude extract (mg/mL). It is calculated according to the following formula:

A (mg/mL) = ((C × V)/P)/1000(7)

where A is total protein concentration (mg/mL crude extract); C—concentration calculated from the calibration curve (mg/g); V—extract volume (ml); P—weight of raw biomass extracted (g).

#### 4.9.2. Catalase (CAT)

The method is based on the reaction of CAT with H_2_O_2_ [56,57]. For CAT assays, 170 μL of buffer (Table 1) and 10 μL of 30% H_2_O_2_ solution (Table 1) were added to 20 μL of crude extract. Analyzes were performed at 240 nm, and the kinetic change in CAT over time (downward trajectory) was monitored every 35 s. Based on the total protein concentration in the tissue, the CAT activity was converted to its activity in the fresh needle biomass:

CAT activity (µmol H_2_O_2_/mg (protein) per min) = ((S_c_ × V_t_)/(0.478 × V_e_ × 39.4))/P_eq_ (8)

where S_c_—slope coefficient; V_t_—total sample volume; V_e_—total extract volume; P_eq_—BSA equivalent based on standard curve. 

#### 4.9.3. Ascorbate Peroxidase (APX)

For APX analyses, 170 μL of ASC solution (Table 1) and 10 μL of 30% H_2_O_2_ solution (Table 1) are added to 20 μL of filtered extract [57,58,59]. Analyzes are performed at 290 nm, and the kinetic change in APX over time (downward trajectory) is monitored every 35 s. Based on the total protein concentration in the tissue, the APX activity was converted to its activity in the fresh needle biomass:

APX activity (µmol ASC/mg (protein) per min) = ((S_c_ × V_t_)/(0.478 × V_e_ × 2.8))/P_eq_ (9)

where S_c_—slope coefficient; V_t_—total sample volume; —total extract volume; P_eq_—BSA equivalent based on standard curve. 

#### 4.9.4. Guaiacol Peroxidase (POX)

For POX assays, 170 μL of solution (Table 1) and 10 μL of 30% H_2_O_2_ (Table 1) are added to 20 μL of filtered extract [56,57]. Analyzes are performed at 430 nm and the kinetic change in POX over time (upward trajectory) is monitored every 35 s. Based on the total protein concentration in the tissue, the POX activity was converted to its activity in the fresh needle biomass:

POX activity (µmol oxidized pyrogallol/mg (protein) per min) = ((S_c_ × V_t_)/(0.478 × V_e_ × 2.46))/P_eq_ (10)

where S_c_—slope coefficient; V_t_—total sample volume; V_e_—total extract volume; P_eq_—BSA equivalent based on standard curve. 

#### 4.9.5. Glutathione Reductase (GR)

The method is based on the oxidation of NADPH by GR-reducing oxidized L-glutathione [57,58,60]. For GR analyses, 20 μL of filtered extract is added to 160 μL of solution (Table 1) and 20 μL of 20 mM oxidized L-Glutathione substrate. Analyzes are performed at a wavelength of 340 nm, and the kinetic change in GR over time (downward trajectory) is monitored every 35 s. Based on the total protein concentration in the tissue, GR activity was converted to its activity in the fresh needle biomass:

GR activity (µmol NADPH/mg (protein) per min) = ((S_c_ × V_t_)/(0.478 × V_e_ × 6.22))/P_eq_ (11)

where S_c_—slope coefficient; V_t_—total sample volume; V_e_—total extract volume; P_eq_—BSA equivalent based on standard curve. 

### 4.10. Statistical Analysis

Group means and standard errors were calculated using Microsoft Excel. Statistical data analysis was performed using the SPSS program (IBM, version 28.0.1.1.). The Kruskal–Wallis H test was used for analysis as a non-parametric alternative to one-way ANOVA. During this test, differences are determined by comparing the mean ranks of groups. A post hoc Dunn’s test was performed to indicate differences between individual pairs. Spearman’s correlation coefficients were calculated for biochemical tests as well, using SPSS. 

## 5. Conclusions

All in all, it was shown that exogenous jasmonic acid application impacts secondary metabolite production in five *Pinus sylvestris* half-sib genetic families; however, no positive effect on seedling growth parameters was observed within the studied time frame (i.e., 6 weeks). Furthermore, jasmonic acid application was shown to specifically increase phenol content and antioxidant enzyme activity in some of the tested pine families, thus inducing systemic resistance. Hence, jasmonic acid application could be utilized as a means to limit pesticide use and help plants with stress response. It is important to take note of the differences based on plant genotype and the concentration of jasmonic acid to be used. 

## Figures and Tables

**Table 1:** Composition of reaction buffers used for antioxidant enzyme analyses.

Analysis	Reaction Solutions
Extraction	Solution: K-phosphate buffer (297 mL), (pH 7.8) [54]; 3 mL of Triton-X; 18 g of polyvinylpolypyrrolidone (PVPP); 1.3209 g of 5 mM ascorbate (ASC) (Chempur, Piekary Śląskie, Poland).
Total protein (PROT)	Biuret reagent: 0.5 mL of 1% CuSO_4_; 0.5 mL of 2% Na-K-tartrate; 50 mL solution consisting of 1 g 2% NaCO_3_ and 0.2 g 0.1 N NaOH.
H_2_O_2_	200 μL 30% H_2_O_2_ added to 10 mL of dH_2_O.
Catalase (CAT)	40 mL of 50 mM K-phosphate buffer (pH 7.0) [54].
Ascorbate peroxidase (APX)	Solution: 40 mL of 50 mM K-phosphate buffer (pH 7.0) [32]; 200 μL of 250 mM ASC Preparation: 0.08806 g of ASC dissolved in 2 mL of dH_2_O.
Guaiacol peroxidase (POX)	Solution: K-phosphate buffer (pH 6.5) [32]; 101 g of pyrogallol (Chempur) dissolved in K-phosphate buffer.
Glutathione reductase (GR)	Solution: 40 mL of 50 mM, HEPES buffer (Sigma Aldrich, St. Louis, MO, USA) (pH 8.0). Preparation: 2.38 g of HEPES dissolved in 100 mL of dH_2_O. 1 mL of 20 mM EDTA. Preparation: 0.584 g of EDTA dissolved in 100 mL of dH_2_O. 2 mL of 5 mM NADPH. Preparation: 0.00834 g of NADPH dissolved in 2 mL of HEPES buffer.Substrate: 0.025 g of substrate oxidized L-glutathione (PanReac Applichem, Darmstadt, Germany) dissolved in 2 mL of dH_2_O

## Data Availability

Data will be made available upon request.
